# Immune Response of Piglets Receiving Mixture of Formic and Propionic Acid Alone or with Either Capric Acid or* Bacillus Licheniformis* after* Escherichia coli* Challenge

**DOI:** 10.1155/2019/6416187

**Published:** 2019-03-25

**Authors:** Chunxiao Ren, Qiqi Zhou, Wutai Guan, Xiaofeng Lin, Yijiang Wang, Hanqing Song, Yinzhi Zhang

**Affiliations:** ^1^Guangdong Province Key Laboratory of Animal Nutrition Control, College of Animal Science, South China Agricultural University, Guangzhou 510642, China; ^2^College of Animal Science and National Engineering Research Center for Breeding Swine Industry, South China Agricultural University, Guangzhou 510642, China

## Abstract

This study aims to evaluate whether diets containing mixture of formic and propionic acid alone or supplemented with either capric acid or* Bacillus Licheniformis *can alleviate immune inflammatory response of piglets challenged with enterotoxigenic* Escherichia coli* (ETEC). A total of 30 weaning pigs were assigned to 5 diets, without additive (CON), with antibiotics (ATB), with 1% organic acid (OA; 64% formic and 25% propionic acid), with OA plus 0.2% capric acid (OA + CRA), and with OA plus 0.02% probiotic (*Bacillus Licheniformis*; OA + PB). After oral challenge with ETEC on day 10, the feces and plasma of all pigs were collected at different time points. Four additive treatments all decreased rectal temperature (RT) at 9 h and fecal scores (FS) at 24 h after challenge (*P* < 0.05), while at 9 h after challenge, inclusion of OA induced a decrease of RT compared with OA + CRA and OA + PB (*P* < 0.05). In plasma, concentration of interleukin (IL)-1*β* was reduced with the addition of ATB and OA at 24 h and 48 h after challenge and it is lower in OA group than OA + CRA group at 24 h after challenge (*P* < 0.05). Diets with ATB, OA, and OA + PB caused a decrease of the concentrations of IL-6 in plasma at 9 h after challenge (*P* < 0.05). The four additives treated piglets showed decreased concentrations of plasma tumor necrosis factor-*α* and interferon-*γ* at 9 h and 24 h after challenge, respectively (*P* < 0.05). In conclusion, OA supplementation alleviated the inflammatory response and reduced diarrhea incidence in piglets challenged with ETEC. However, no further improvements were observed when OA supplemented with CRA or probiotics.

## 1. Introduction

Weaning pigs frequently appear postweaning diarrhea, due to the insufficient quantities of gastric acid resulting in a high pH value in stomach and a high risk of enterotoxigenic* Escherichia coli* (ETEC) infection [1], which leads a lasting detrimental effect on performance [2]. Antibiotics growth promoters have been used in the weaning piglets' diets for many years and have been proved to be an effective way of attenuating post-weaning diarrhea and improving growth performance of piglets [3], while it was reported that the antibiotics used in livestock accounted for a substantial portion of total antibiotics production [4]. However, the long-term use of antibiotics has accelerated the biological evolution of microorganisms, resulting in the emergence of many drug-resistant microorganisms that threaten human health [5]. Therefore, finding alternatives to antibiotics in livestock production to reduce the risk of bacterial resistance to human is urgently needed.

Organic acids, i.e., formic acid and propionic acid, have long been added to weaning pig diets for making up the insufficient quantities of gastric acid to activate pepsin and inhibit the proliferation of pathogens in gastrointestinal tract of piglets [6]. Medium-chain fatty acids (MCFAs), i.e., caproic acid, caprylic acid, capric acid, and lauric acid, exhibit broad-spectrum antibacterial properties [7]. Studies have shown that organic acids and MCFAs have a synergistic effect of killing diarrhea causing pathogens [8, 9]. Probiotics such as* Bacillus Licheniformis* have also been widely promoted as alternatives to the antibiotics.* Bacillus Licheniformis* is able to secrete digestive enzymes, tolerate gastric acid, and compete with pathogens for colonization sites and nutrients [10]. However, there was limited information on whether combinations of formic/propionic acid and capric acid or* Bacillus Licheniformis* could alleviate the intestinal of piglets and whether synergistic action between them exists in vivo. Thus, the effect of dietary supplementation with formic/propionic and its combinations with capric acid or* Bacillus Licheniformis* on the immune response of weaning pigs challenged with ETEC K88 was determined in present study.

## 2. Materials and Methods

### 2.1. Experimental Diets and Feeding Regime

The experimental basal diet was formulated to meet the nutrients requirements for a 6 to 10 kg pig (NRC, 2012) and its compositions and nutrient profile were shown in Table 1. Experimental diets are as follows: (1) basal diet without other supplements as a negative control (CON); (2) basal diet with antibiotics (50 mg/kg quinocetone, 75 mg/kg chlortetracycline, 50 mg/kg kitasamycin; ATB); (3) basal diet plus 1% mixture of 64% formic and 25% propionic acid (Lupro-Cid; BASF, Germany; OA); (4) basal diet plus 1% OA and 0.2% capric acid (obtained from sigma-aldrich; OA + CRA); (5) basal diet plus 1% OA and 0.02% probiotic (*Bacillus Licheniformis*, 1.65 × 10^10^ viable spores per gram, 3.3 × 10^6^ viable spores of* Bacillus Licheniformis* per gram of feed; OA + PB). During the whole period of the experiment, the piglets were ad libitum to the feed and water and diets were offered to pigs as mash.

### 2.2. Animals and Housing

A total of 30 male piglets (Duroc × Landrace × Yorkshire, initial weight 7.9 ± 0.5 kg) were weighed and randomly allocated to dietary 5 treatments with 6 replicate pens per treatment and 1 pig per pen. The animal use and care protocol were approved by the South China Agricultural University Animal Care and Use Committee.

The temperature of the room was maintained at 30 ± 1°C. After placement, pigs were allowed a 6-d adaptation period to acclimate to their new surroundings and feed. During the adaptation period, the piglets showed no signs of diarrhea, skin wounds, or obvious inflammation, which suggested that pigs were healthy and fit for the experiment.

### 2.3. Bacteria Preparation, Oral Challenge, and Fecal Microbial Analysis

*Escherichia coli* K88 (CVCC225) was purchased from the Chinese Veterinary Medicine Collection Center. The* Escherichia coli* K88 was confirmed by polymerase chain reaction (PCR) genotyping as possessing the genes necessary for heat labile enterotoxin (LT), heat stable enterotoxin (ST), and F4 fimbrial antigen using published primers [11, 12]. After an adaption period of 6 days, the piglets were fed corresponding experimental diets for 9 days and orally challenged with 10 mL of 5 × 10^9^ CFU/mL ETEC K88 via a syringe on the 10th day. Fresh faeces were collected and weighed at 0, 24, and 48 h after ETEC K88 challenge and the fecal pH values were determined using pH meter (Testo 205). Total coliforms were quantified by 10-fold gradient dilution method using eosin methyl blue agar and the plates were incubated aerobically at 37°C for 24 to 48 h [13]. For* Lactobacillus* spp. counts, the serially diluted samples were quantified with Man, Rogosa, and Sharpe agar and incubated for 48 h at 37°C under anaerobic condition [14].

### 2.4. Rectal Temperature and Diarrhea Assessment

Diarrhea assessment was determined according to fecal scores (FS) system reported by Marquardt et al. [15]. Occurrence and severity of diarrhea were monitored at 0, 9, 24, 48, 96, and 168 h after challenge and assessed as follows: 0; normal; 1; soft feces; 2: mild diarrhea; 3: severe diarrhea by 1 trained personnel with no prior knowledge of the dietary treatment allocation. The rectal temperature (RT) of the piglets was measured by rectal thermometer (Omron MC-347) at 0, 9, 24, 33, 48, 96, and 168 h after ETEC K88 challenge.

### 2.5. Plasma Parameter Analysis

Blood samples were collected via anterior vena cava puncture at 0, 9, 24, 48, 96, and 168 h after challenge into tubes containing EDTA. To obtain the plasma samples, blood samples were centrifuged at 1000 × g for 10 min and then stored at −20°C and analyzed within one month. Plasma concentrations of interleukin (IL)-1*β*, IL-6, interferon-*γ* (IFN-*γ*), and tumor necrosis factor-*α* (TNF-*α*) were determined using a porcine-specific multiplex assay (Millipore Multiplex; Billerica, MA, #PCYTMAG-23K-05) according to Vieira‐Potter et al. [16]. Immunoglobulin G (IgG) in the plasma was determined using a commercially available porcine IgG Elisa kit (Huamei, Wuhan). All assays were run in duplicate.

### 2.6. Statistical Analysis

Data were analyzed as a completely randomized design by using GLM procedures with SPSS (SPSS Software Release 22, IBM Inc., USA). For every assay analysis, a generalized randomized design with repeated measures over time on each experimental unit (individual pig) was followed. Comparisons between dietary treatments and sampling times were made when a significance was found using LSD procedure. All data were presented as least squares means ± SEM.

## 3. Results

### 3.1. Rectal Temperature and Fecal Scores

**Figure 1:**
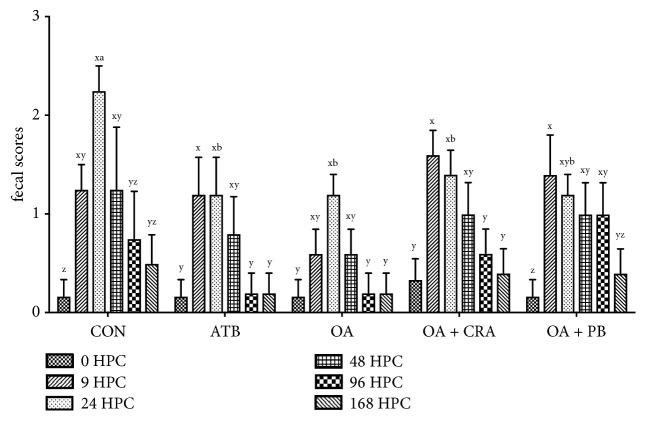
Effects of organic acid or organic acid supplemented with either capric acid or* Bacillus Licheniformis* on fecal scores of pigs challenged with enterotoxigenic* Escherichia coli* (ETEC). CON = basal diet; ATB = basal diet plus antibiotics; OA = basal diet plus 1% organic acid; OA + CRA = basal diet plus 1% organic acid + 0.2% capric; OA + PB = basal diet plus 1% organic acid + 0.02% probiotic (3.3 × 10^6^ viable spores of* Bacillus Licheniformis* per gram of feed). Values are least squares means ± SEM. ^x-z^ Within per treatment, value at different time points without a common superscript differs (*P* < 0.05); ^a-b^ within per time point, value with different treatments without a common superscript differs (*P* < 0.05).

**Figure 2:**
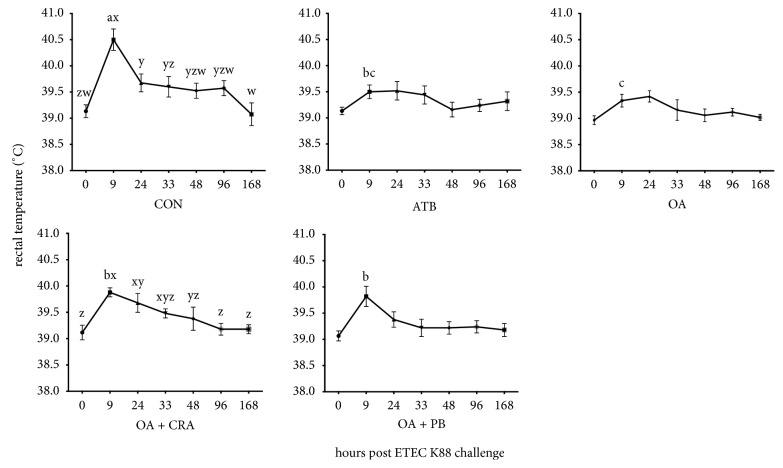
Effects of organic acid or organic acid supplemented with either capric acid or* Bacillus Licheniformis* on rectal temperature of pigs challenged with enterotoxigenic* Escherichia coli *(ETEC). CON = basal diet; ATB = basal diet plus antibiotics; OA = basal diet plus 1% organic acid; OA + CRA = basal diet plus 1% organic acid + 0.2% capric; OA + PB = basal diet plus 1% organic acid + 0.02% probiotic (3.3 × 10^6^ viable spores of* Bacillus Licheniformis* per gram of feed). Values are least squares means ± SEM. ^w-z^ Within per treatment, value at different time points without a common superscript differs (*P* < 0.05); ^a-b^ within per time point, value with different treatments without a common superscript differs (*P* < 0.05).

Overall, after challenge, the FS increased in all groups (*P* < 0.05) and the control pigs had higher FS than pigs in other four treatment groups at 24 h after challenge (*P* < 0.05) (Figure 1). The ETEC challenge caused an increased RT of piglets in CON and OA + CRA groups (*P* < 0.05) with higher RT at 24 h than 0 h after challenge in these two groups (*P* < 0.05) (Figure 2). The piglets showed lower RT when they fed diets with additives, and OA treated piglets had lower RT compared with OA + CRA or OA + PB at 9 h after challenge (*P* < 0.05).

### 3.2. Inflammatory Cytokines and Immunoglobulin G in Plasma

After challenge, higher plasma IL-1*β* concentration was appeared at 9, 24, and 48 h than 0 h in CON group (*P* < 0.05), and higher plasma IL-6 concentrations were appeared at 9 h than 0 h in CON group (*P* < 0.05) (Tables 2 and 3). With the addition of ATB and OA, plasma IL-1*β* concentration was decreased at 24 and 48 h after challenge (*P* < 0.05), while at 24 h after challenge OA supplementation decreased plasma IL-1*β* concentration compared with OA + CRA (*P* < 0.05). Pigs receiving ATB, OA, and OA + PB diets decreased IL-6 concentration in plasma at 9 h after challenge compared with CON group (*P* < 0.05).

After challenge, plasma TNF-*α* concentrations significantly increased in CON, OA, and OA + CRA groups (*P* < 0.05) and plasma IFN-*γ* concentrations significantly increased in CON and OA + CRA groups (*P* < 0.05) (Tables 4 and 5). The higher plasma TNF-*α* and IFN-*γ* concentrations were observed in the pigs of CON groups than the pigs of other four treatment groups at 9 and 24 h after challenge, respectively (*P* < 0.05). Plasma IgG concentration was significantly increased in OA + CRA group after challenge (*P* < 0.05) and did not differ significantly among the five treatment groups (*P* > 0.05) (Table 6).

### 3.3. Microbial Populations in Feces

There was no effect of treatment on fecal pH values and total coliforms and* Lactobacillus* counts observed at 0, 24, and 48 h after challenge (*P* > 0.05) (Table 7).

## 4. Discussion

Oral challenge with ETEC has been widely used as model to screen out effective antibiotic substitutes that protect piglets from suffering postweaning diarrhea [17], which causes impaired growth performance and high mortality [18]. The lipopolysaccharide of ETEC can activate the nuclear factor-*κ*B pathway through toll like receptor 4, leading to the occurrence of intestinal epithelial cell inflammation [19], while the LT and ST produced by ETEC K88 can cause diarrhea in piglets through the cystic fibrosis transmembrane regulator of intestinal epithelial cells [20]. In this study, fecal scores were used to evaluate intensity of diarrhea in piglets according to methods previously reported [15, 21]. After challenge, the fecal scores of piglets increased in each treatment group, and piglets receiving ATB, OA, OA + CRA, and OA + PB diets all exhibited lower fecal scores compared with CON group at 24 h after challenge. Short chain fatty acids have a broad-spectrum bactericidal capacity against gram negative bacteria and can kill pathogens at low pH [22, 23]. Therefore, adding short chain fatty acid in the diet can form a line of defense in the low pH condition of stomach to kill pathogens, thereby, weakening the pathogen's damage to the small intestine of piglets. Additionally, expression of virulence gene was suppressed when pathogens were grown in medium supplemented with subminimal inhibition concentration of short and medium chain fatty acids [24–26]. Dietary inclusion of formic/propionic mixture may reduce the harm of ETEC to the intestines and then decrease the incidence of piglet diarrhea.

In the present study, no additional effects on the fecal scores of piglets were observed when organic acids combined with medium-chain fatty acids. It has been reported that short and medium-chain fatty acids have a synergistic bactericidal effect in vitro [8, 9]; nevertheless, the antimicrobial ability of capric acid was not only to* Escherichia coli* but also to* Lactobacillus* (conformed in vitro in our lab, but data has not been publicly reported); when they used together, they may influence the* Lactobacillus* counts in the gut of the piglets. However, some researches showed that inclusion of OAs and MCFAs in the piglets' diets enriched* lactobacillus* in ileal and rectal content [27], MCFAs at low dietary levels increased* lactobacillus johnsonii* and* lactobacillus amylovorus* counts in gastric contents [28], and dietary 0.15% MCFAs induced minor changes in the gastrointestinal microbiota [29]. The different gastrointestinal microbiota response to MCFAs may be due to the dosage and species of MCFAs in the diets, in which the lower dosage MCFAs performed beneficial gut flora modifier, and the optimal dosage of MCFAs in the piglets' diet needs further exploration.* Bacillus Licheniformis* was also reported to control postweaning diarrhea syndrome of piglets [30]. However, no additional effects on the fecal scores of piglets were observed when organic acids combined with* Bacillus Licheniformis*. The reports about the combination of probiotic and organic acids in the piglets' diets were limited, and Wolfenden et al. [31] reported combination treatment with organic acid and* lactobacillus* was more effective than individual treatment for* Salmonella* reduction in chicks. No better improvements when organic acid was supplemented with probiotic in the present study may be due to the different probiotic strains, and the combined effects of organic acids and different probiotics may vary.

Inflammation of intestinal epithelial cells was induced by activating the nuclear factor-*κ*B pathway by lipopolysaccharide, LT, and ST of ETEC, which increased the animal body temperature [21]. Rectal temperature is a sensitive indicator of intestinal inflammation in oral ETEC challenge model, and short-term temperature rises after challenge had been appeared in other ETEC challenge reports [18]. Our results also implied that RT of piglets were increased after challenge with ETEC, after reaching the peak value, and the RT of piglets in all treatment groups began to gradually decrease to normal physiological value within 168 hours. At 9 h after challenge, piglets fed OA and ATB diets decreased RT relative to CON diet, which was observed in other trials of some effective antibiotic substitutes [21, 32, 33]. In the present study, 1% of formic/propionic acid mixture mitigated fever of piglets induced by ETEC challenge to a similar extent as the antibiotic treatment.

During the process of inflammation, IL-1*β* and IL-6 in blood can produce heat and cause an increase in body temperature. Compared with CON pigs, those fed OA decreased plasma IL-6 concentration at 9 h after challenge, which was consistent with the RT displayed in CON and OA groups. Addition of 1% of an organic acid blend to feeds of ETEC challenged piglets suppressed inflammation therefore decreasing RT after challenge to a level on par with the antibiotic treatment group. Similarly, Zhang et al. [34] reported that IL-1*β* level in serum was decreased at 6 h after ETEC K88 challenge in piglets orally inoculated with* Lactobacillus rhamnosus GG*, while Kiarie et al. [35] reported that piglets fed diets containing either egg yolk antibodies or nonstarch polysaccharide hydrolysis products decreased serum IL-6 concentration at 6 h after ETEC K88 challenge. In these studies,* Lactobacillus rhamnosus GG* and egg yolk appeared to alleviate the pronounced acute inflammatory response induced by ETEC K88 in piglets. These studies demonstrated that this formic/propionic acid mixture can also be used as an effective antibiotic substitute to relieve inflammation induced by ETEC K88.

Plasma TNF-*α* was measured as indicators of systemic pro-inflammation response and primarily secreted by activated phagocytic cells during the period of an acute enteric disease. The depression of circulating TNF-*α* in OA, OA + CRA, and OA + PB group compared to CON group was observed at 9 h after challenge, and this reduction was observed in the ETEC challenge pigs of other reports [34]. Consistent decline in circulating IFN-*γ* was also observed at 24 h after challenge. Interferon-*γ* contributes to T helper cell 1 differentiation and bridges the innate and specific immune response. It was possible that piglets receiving OA, OA + CRA, and OA + PB not only relieved initial immune response but also alleviated cellular immune responses after ETEC challenge. The changes of TNF-*α* and IFN-*γ* also reflected that no additional mitigation was observed when OA plus either CRA or PB. Although marked synergistic bactericidal effect of organic acids in combination with MCFAs was reported in vitro [8, 9] and organic acids and MCFAs had an interaction in reducing counts of Enterobacteria in the stomach [29]. Nevertheless, pigs fed diets containing 1% of a formic/propionic acid mixture performed well and the overall level of infection challenge was most likely low. Therefore, testing the capric or* Bacillus Licheniformis *under lower doses of organic acid or under greater disease challenge may be warranted.

After challenge, IgG in plasma of piglets increased with time, indicating that IgG mediated humoral immunity and exerted antibacterial effects in the body of piglets. However, no significant difference was found between each treatment group. Before and after the challenge, no significant difference was found in the fecal pH values and total* E. coli* and* Lactobacillus* counts. This may be due to the fact that liquid organic acids would prevent the passage of pathogens and regulate the microbes in the stomach and proximal segments of intestine [36]. It is possible that organic acids were absorbed in mid and posterior segment of the small intestine [37], and they had little effects on the number of microbes in posterior segment of intestinal and feces.

## 5. Conclusion

Overall, administrating 1% of a formic/propionic acid mixture alleviated the inflammatory response of weaning pigs challenged with ETEC K88 and then reduced febrile and diarrhea. This was comparable to the reference group supplemented with antibiotics commonly used by pig producers in weaning feed to improve performance and to prevent enteric diseases. Therefore, this study suggests that the use of this formic/propionic acid mixture at 1% in weaning pig diets can be considered a valid alternative to antibiotics, at least with regard to ETEC challenge. However, no further improvements were observed when the organic acid blend was supplemented in conjunction with either capric or* Bacillus Licheniformis.*

## Figures and Tables

**Table 1:** Compositions of basal diet and nutrient profile, as-fed basal.

Ingredient composition	Content (%)	Calculated nutrient profile	Content
Corn	47.25	Digestible energy (MJ/kg)	15.05
Wheat powder	7.50	CP (%)	19.20
Whey	7.50	Crude fat (%)	8.03
Sucrose	2.50	Crude fiber (%)	1.76
Glucose	1.25	Ash (%)	3.01
Soybean Meal	8.50	Calcium (%)	0.77
Extruded soybean	10.00	Phosphorus (%)	0.53
Fish meal	7.00	Available phosphorus (%)	0.34
Soybean oil	2.00	Lysine (%)	1.35
Yeast extract	2.50	Methionine+ Cysteine (%)	0.74
Choline chloride (50%)	0.10	Threonine (%)	0.79
Calcium hydrophosphate	0.38	Tryptophan (%)	0.22
Limestone	0.8		
Salt	0.30		
L-Lysine HCl	0.50		
DL-Methionine	0.22		
L-Threonine	0.25		
L-Tryptophan	0.02		
L-Valine	0.1		
Vitamin premix^1^	0.035		
Mineral premix^2^	0.23		
Unite bran	1.065		
Total	100.00		

^1^Supplied per kilogram of diet: 11375 IU of vitamin A, 3500 IU of vitamin D_3_, 26.3 IU of vitamin E, 3.5 mg of vitamin of K_3_, 3.5 mg of vitamin B_1_, 8.8 mg of riboflavin, 5.4 mg of vitamin B_6_, 0.03 mg of vitamin B_12_, 17.5 mg of pantothenic acid, and 35.0 mg of niacin; 1.75 mg of folacin and 0.14 mg of biotin.

^2^Supplied per kilogram of diet: 64.4mg of Cu (cupric glycinate), 165.4mg of Fe (iron glycine), 47.8 mg of Mn (manganese glycinate), 47.8mg of Zn (zinc glycinate), 0.54 mg of Se (yeast selenium), 0.68 mg of I (calcium iodate), and 0.1 mg of Co (cobaltous sulfate).

**Table 2:** Effect of organic acid or organic acid supplemented with either capric acid or *Bacillus Licheniformis* on interleukine-1*β* (pg/mL) concentration in plasma of pigs challenged with enterotoxigenic *Escherichia coli*.

Time, h	Treatments					SEM	*P*-value
	CON	ATB	OA	OA + CRA	OA + PB		
0 HPC^1^	900^z^	631	877	882	902	53	0.444
9 HPC	1351^xy^	821	1075	1141	1175	85	0.438
24 HPC	1605^xa^	711^b^	860^b^	1359^a^	1252^ab^	92	0.005
48 HPC	1420^xa^	774^c^	864^bc^	1299^ab^	1024^abc^	80	0.035
96 HPC	1255^xyz^	740	751	1173	914	81	0.144
168 HPC	934^yz^	769	741	959	826	55	0.678
SEM	77	57	54	64	66		
*P*-value	0.023	0.959	0.578	0.201	0.406		

CON = basal diet; ATB = basal diet plus antibiotics; OA = basal diet plus 1% organic acid; OA + CRA = basal diet plus 1% organic acid + 0.2% capric; OA + PB = basal diet plus 1% organic acid + 0.02% probiotic (3.3×10^6^ viable spores of *Bacillus Licheniformis* per gram of feed).

^1^ HPC: hours after challenge.

^x-z^ Within per treatment, values at different time points without a common superscript differ significantly at *P *< 0.05; ^a-b^ within per time point, values with different treatments without a common superscript differ significantly at *P *< 0.05.

**Table 3:** Effect of organic acid or organic acid supplemented with either capric acid or *Bacillus Licheniformis* on interleukine-6 (pg/mL) concentration in plasma of pigs challenged with enterotoxigenic *Escherichia coli*.

Time, h	Treatments					SEM	*P*-value
	CON	ATB	OA	OA + CRA	OA + PB		
0 HPC^1^	280.2^y^	283.8	294.4	304.2	258.7	12.8	0.860
9 HPC	480.8^xa^	369.8^b^	358.7^b^	423.1^ab^	372.5^b^	14.6	0.045
24 HPC	395.4^xy^	315.1	319.3	386.5	324.5	19.3	0.556
48 HPC	350.2^y^	284.6	325.3	312.8	307.1	17.7	0.872
96 HPC	303.0^y^	268.1	306.2	323.2	295.0	17.4	0.911
168 HPC	286.2^y^	274.5	284.4	292.9	297.0	15.1	0.993
SEM	20.9	13.9	14.1	15.8	16.2		
*P*-value	0.022	0.304	0.749	0.095	0.483		

CON = basal diet; ATB = basal diet plus antibiotics; OA = basal diet plus 1% organic acid; OA + CRA = basal diet plus 1% organic acid + 0.2% capric; OA + PB = basal diet plus 1% organic acid + 0.02% probiotic (3.3×10^6^ viable spores of *Bacillus Licheniformis* per gram of feed).

^1^ HPC: hours after challenge.

^x-z^ Within per treatment, values at different time points without a common superscript differ significantly at *P *< 0.05; ^a-b^ within per time point, values with different treatments without a common superscript differ significantly at *P *< 0.05.

**Table 4:** Effect of organic acid or organic acid supplemented with either capric acid or *Bacillus Licheniformis* on tumor necrosis factor-*α* (pg/mL) concentration in plasma of pigs challenged with enterotoxigenic *Escherichia coli*.

Time, h	Treatments					SEM	*P*-value
	CON	ATB	OA	OA + CRA	OA + PB		
0 HPC^1^	160.9^y^	119.3	159.5^y^	172.2^z^	139.2	12.7	0.727
9 HPC	475.4^xa^	213.7^b^	288.3^xb^	306.2^xyb^	226.6^b^	24.7	0.003
24 HPC	297.3^xy^	153.4	239.8^xy^	328.5^x^	259.1	26.2	0.267
48 HPC	316.1^xy^	163.2	237.5^xy^	202.1^xyz^	189.1	22.1	0.276
96 HPC	250.9^y^	173.2	165.0^y^	190.2^yz^	191.2	17.8	0.672
168 HPC	166.9^y^	127.9	146.2^y^	139.0^z^	168.3	15.7	0.927
SEM	33.1	11.0	15.9	20.7	15.2		
*P*-value	0.042	0.144	0.039	0.031	0.243		

CON = basal diet; ATB = basal diet plus antibiotics; OA = basal diet plus 1% organic acid; OA + CRA = basal diet plus 1% organic acid + 0.2% capric; OA + PB = basal diet plus 1% organic acid + 0.02% probiotic (3.3×10^6^ viable spores of *Bacillus Licheniformis* per gram of feed).

^1^ HPC: hours after challenge.

^x-z^ Within per treatment, values at different time points without a common superscript differ significantly at *P *< 0.05; ^a-b^ within per time point, values with different treatments without a common superscript differ significantly at *P *< 0.05.

**Table 5:** Effect of organic acid or organic acid supplemented with either capric acid or *Bacillus Licheniformis* on interferon-*γ* (ng/mL) concentration in plasma of pigs challenged with enterotoxigenic *Escherichia coli*.

Time, h	Treatments					SEM	*P*-value
	CON	ATB	OA	OA + CRA	OA + PB		
0 HPC^1^	13.72^z^	14.32	13.01	13.23^y^	12.25	0.85	0.962
9 HPC	24.54^y^	21.40	15.19	17.79^xy^	22.70	1.46	0.272
24 HPC	35.26^xa^	24.88^b^	17.02^b^	23.51^xb^	20.77^b^	1.89	0.033
48 HPC	24.29^y^	16.60	15.70	18.31^xy^	18.89	1.31	0.343
96 HPC	22.08^y^	15.38	14.69	14.50^y^	13.62	1.34	0.352
168 HPC	16.54^yz^	14.56	12.51	14.25^y^	12.36	1.11	0.809
SEM	1.72	1.74	0.55	1.03	1.32		
*P*-value	<0.001	0.427	0.159	0.027	0.053		

CON = basal diet; ATB = basal diet plus antibiotics; OA = basal diet plus 1% organic acid; OA + CRA = basal diet plus 1% organic acid + 0.2% capric; OA + PB = basal diet plus 1% organic acid + 0.02% probiotic (3.3×10^6^ viable spores of *Bacillus Licheniformis* per gram of feed).

^1^ HPC: hours after challenge.

^x-z^ Within per treatment, values at different time points without a common superscript differ significantly at *P *< 0.05; ^a-b^ within per time point, values with different treatments without a common superscript differ significantly at *P *< 0.05.

**Table 6:** Effect of organic acid or organic acid supplemented with either capric acid or *Bacillus Licheniformis* on Immunoglobulin G (*μ*g/mL) concentration in plasma of pigs challenged with enterotoxigenic *Escherichia coli*.

Time, h	Treatments					SEM	*P*-value
	CON	ATB	OA	OA+ CRA	OA + PB		
0 HPC^1^	1210	1373	1461	1219^w^	1569	104	0.801
9 HPC	1544	1643	1683	1458^zw^	1487	137	0.985
24 HPC	1651	1414	1714	1604^yzw^	1726	98	0.871
48 HPC	1867	1450	1414	1741^yz^	1887	121	0.625
96 HPC	2723	1798	1899	2072^xy^	2131	111	0.099
168 HPC	2482	2039	2080	2290^x^	2196	104	0.743
SEM	174	104	86	92	123		
*P*-value	0.061	0.408	0.198	0.001	0.479		

CON = basal diet; ATB = basal diet plus antibiotics; OA = basal diet plus 1% organic acid; OA + CRA = basal diet plus 1% organic acid + 0.2% capric; OA + PB = basal diet plus 1% organic acid + 0.02% probiotic (3.3×10^6^ viable spores of *Bacillus Licheniformis* per gram of feed).

^1^ HPC: hours after challenge.

^w-z^ Within per treatment, value at different time points without a common superscript differs(*P*<0.05); ^a-b^ within per time point, value with different treatments without a common superscript differs (*P*<0.05).

**Table 7:** Effect of organic acid or organic acid supplemented with either capric acid or *Bacillus Licheniformis* on fecal pH values and microbial counts (log_10_ CFU/g) of piglets challenged with enterotoxigenic *Escherichia coli*.

Item	Treatments					SEM	*P*-value
	CON	ATB	OA	OA + CRA	OA + PB		
0 HPC^1^							
pH	6.93	6.76	6.71	6.74	6.65	0.06	0.700
Total coliforms	7.32	7.15	7.48	7.22	7.04	0.11	0.777
*Lactobacillus*	8.14	8.58	8.59	8.20	9.01	0.15	0.410
24 HPC							
pH	7.60	7.39	7.28	7.26	7.39	0.05	0.331
Total coliforms	7.71	7.28	7.17	7.13	6.98	0.12	0.489
*Lactobacillus*	8.15	8.33	8.41	8.37	7.93	0.15	0.867
48 HPC							
pH	7.47	7.41	7.23	7.29	7.20	0.04	0.298
Total coliforms	7.26	7.12	7.19	7.08	6.67	0.17	0.872
*Lactobacillus*	8.71	8.63	8.43	8.10	8.39	0.15	0.833

CON = basal diet; ATB = basal diet plus antibiotics; OA = basal diet plus 1% organic acid; OA + CRA = basal diet plus 1% organic acid + 0.2% capric; OA + PB = basal diet plus 1% organic acid + 0.02% probiotic (3.3×10^6^ viable spores of *Bacillus Licheniformis* per gram of feed).

^1^ HPC: hours after challenge.

## Data Availability

The data used to support the findings of this study are included within the article.
